# New Appliances and Things Medical

**Published:** 1897-09-04

**Authors:** 


### NEW APPLIANCES AND THINGS MEDICAL.

[We shall be glad to receive, at our Office, 28 & 29, Southampton Street, Strand, London, W.C., from the manufacturers, specimens of all new preparations and appliances which may be brought out from time to time.]

### SCOTCH WHISKY, SCOTSMAN BLEND.

(J. H. Dewar, 47, Rose Street, Glasgow.)

We have recently received three samples of whisky from the above Glasgow firm, 6, 10, and 15 years' old respectively. It is specially claimed for this spirit that it is made exclusively from malt, and that it contains no grain or potato alcohol. Our investigations show that all three spirits are practically of the same alcoholic strength; the percentage of extractives is higher in the more matured whisky, and increases in a ratio corresponding to the age. We have no hesitation in recommending the Scotsman blend as a whisky of excellent quality. Its mellowness and agreeable flavour will certainly secure for it a popular position among the ever-increasing roll of whisky drinkers.

### BOVRIL STAMNOIDS.

(Bovril, Limited, 30, Farringdon Street, E.C.)

In the form of small round lozenges the Bovril Company have succeeded in condensing a truly remarkable amount of nitrogenous food. They are readily soluble in the mouth, by no means disagreeable to the palate, and from personal experience we can testify that they are remarkably sustaining and refreshing. Their ready solubility ensures for them rapid absorption from the stomach, so that for invalids, athletes, and others who require a large amount of nourishment in the most physiologically economical form, we believe that up to the present nothing to approach Bovril Stamnoids has been put upon the market. The stamnoids may be procured in small bottles and carried with no inconvenience in the waistcoat pocket.

